# Report of a Case of Splenectomy

**Published:** 1905-07

**Authors:** D. L. Peeples

**Affiliations:** Navasota, Texas, Surgeon and Major T. N. G.


For Texas Medical Journal.

### Report of a Case of Splenectomy.

BY D. L. PEEPLES, M. D., NAVASOTA, TEXAS,
Surgeon and Major T. N. G.

Mrs. Wm. J. F. J., aged 30, white, confined October 1, 1904, this being her third confinement, the first child having died with inanition, though had the same characteristic eruptions as the others, which were various types of inpetigo literally covering them from head to foot, including the upper extremities and lasting no brief period. However, after no inconsiderable period of treatment the cutaneous dusturbances gradually yielded to treatment. During the last pregnancy the mother's blood was materially impoverished, she was anaemic and suffered with general debility. After delivery a tardy recovery obtained, however emaciation, anorexia and general physical debility continued. Clinical history unobtainable or negative, except having lived in a malarial district all of her life and being susceptible to various types of the prevailing fevers due to the plasmodium or hematozoan. She lived in or near the Brazos and Navasota river bottoms, and in observing no preventive measures she suffered with the above types of fever for ten years. Her physician, Dr. J. F. Eaves, discovered a rapidly growing tumor practically covering the abdominal cavity in its entirety. Therefore I was called in consultation to determine on some course of surgical interference. In consequence, we concurred in its extirpation. This was December 16, 1904. The examination afforded the opportunity of outlining the tumor extending from the original position of the spleen across to the outer and under surfaces of the liver and from the gastric region to the os pubis, thereby covering the entire cavity as stated above. The enormity of the spleen would not infrequently induce paroxysms of dyspnea, and more especially when in the recumbent posture. On account of unfavorable weather, unhygienic surroundings and the difficult problem of maintaining a sufficiency of heat in the home, with necessary comforts under such circumstances, the operation was deferred until 4 p. m. January 1, 1905. Then, the patient being prepared with the usual antiseptic precautions (the absence of nurses and assistants was very observable), I proceeded by making an incision from the umbilicus down to the pubes between the two rectus muscles, giving me the opportunity to release the mass from various adhesions and extirpating the tumor through the wound. Such a large incision afforded greater means for evisceration, though this was not countenanced, as the intestines were carefully enclosed in sterile gauze in a hot normal saline solution, which proved very satisfactory. The organ being lifted out from the cavity, I then clamped the ingress and egress vessels of the spleen with long forceps and began separating each in its turn, ligating the organ with large cat gut ligatures, then severing the attachments of the organ with long scissors, upon which an unimaginable hemorrhage ensued of such magnitude and terrific gravity that apparently my astonishment and insuavity would be almost inadequate to picture. Though exercising emergency efforts, composure and concealed amazement, I succeeded in clearing away the blood and identifying the source of its origin to my great gratification, and found the stump not even oozing; therefore the great gush came forth from the engorged foreign body. The organ being now extirpated, the cavity well cleansed with a saline solution, the viscera returned, the wound agglutinated, including the peritoneum, an appropriate dressing applied, and only a secondary one was necessary as healing rapidly occurred by first intention. The patient was up attending to her usual household duties on January 28, 1905. Her weight prior to her illness was 130 pounds; at the time of the operation she weighed 106 pounds, but on April 12th she weighed 132 pounds. She had an uninterrupted convalescence, with no sequellæ of any character. I have recently obtained information of her excellent health.

